# Impacts of Building Features on the Cooling Effect of Vegetation in Community-Based MicroClimate: Recognition, Measurement and Simulation from a Case Study of Beijing

**DOI:** 10.3390/ijerph17238915

**Published:** 2020-11-30

**Authors:** Wei Chen, Jianjun Zhang, Xuelian Shi, Shidong Liu

**Affiliations:** 1 School of Land Science and Technology, China University of Geosciences, Beijing 100083, China; chenwei_harvey1214@163.com (W.C.); Shaelyn_shi@126.com (X.S.); sheldon_liu1235@126.com (S.L.); 2 Key Laboratory of Land Consolidation and Rehabilitation, Ministry of Natural Resources, Beijing 100083, China

**Keywords:** remote sensing, urban thermal environment, land surface feature

## Abstract

Due to the accumulation of heat, the urban environment and human health are threatened. Land surface cover has effects on the thermal environment; nevertheless, the effects of land surface features and spatial patterns remain poorly known in a community-based microclimate. This study quantified and verified the impacts of normalized difference vegetation index (NDVI) on land surface temperature (LST) (K, the slope of the trend line of a linear regression between NDVI and LST) in different building density by using building outline and Landsat 8 satellite imagery. Comparing the cooling effect and distribution of vegetation showed that the vegetative cover had a cooling effect on LST, characterized by synchronous change, and building density had a significant impact on the cooling effect of vegetation. Through identification and simulation, it was found that the key factor is the wind speed between the buildings because, in different building densities, the wind speed was different, and studies had shown that when the building density was between 0.35 and 0.50, the wind speed between buildings was higher, resulting in a better cooling effect of vegetation. This conclusion has important reference significance for urban planning and mitigating the impact of the thermal environment on human health.

## 1. Introduction

In recent decades, urbanization and industrialization have, without a doubt, become the major driving force of global economic prosperity, especially in developing countries. A great number of people and related human activities are transferred and then tailored to urban settings. Correspondingly, surface roughness is of increasing complexity, which is commonly caused by spreading built-up areas and intensifying buildings and structures [1,2,3]. Such change of surface roughness regulates or even restructures local meteorological conditions, i.e., cumulative urban heat from wildness to urban areas. Quite some work in this direction has been carried out by Ricci et al. [4,5,6] CFD simulations and full-scale measurements. The authors have confirmed and proven how the surface roughness, the large-scale forcing (inflow conditions) and local scale forcing (e.g., buildings) can strongly affect the wind effects inside an urban environment. The phenomenon that increasing people have been exposed to heat environments and suffered from extreme heat has aroused concerns of governments around the world [7]. Affected by the thermal environment, the morbidity and mortality of heat stress and heat-related diseases have increased [8,9,10]. The sustainable development of the environment is also affected by the increase of factors with high photochemical reaction levels, such as the ozone [11,12]. Therefore, research on mitigating urban heat islands has become the focus of scholarly attention. Studying the formation mechanism of the thermal environment is the prerequisite for mitigating urban heat islands.

Affected by many factors, the formation of urban thermal environments is complicated. Absorbing and releasing solar energy, a large amount of urban heat comes from solar energy. Land use and land cover changes caused by urbanization show different absorption and albedo for solar energy due to different materials [13,14]. The land surface temperature is high in bare land, factories, airports, workshops, and low in places with much vegetation. Moreover, there are a large amount of heat accumulations in places with dense population distribution, serious pollution, and traffic congestion [15,16]. Various land cover indices that use remote sensing technologies for extraction have different effects on LST [17,18]. Land cover changes directly affect the accumulation and dispersal of heat on the city surface. Further, the effects of land composition on LST are stronger than other explanatory variables [19,20]. The two most researched factors are vegetation and buildings, as they are each important parts of a city and the main powers to regulate the urban thermal environment [7,21,22,23,24]. Using high albedo materials, pavements, green vegetation, green roofs, urban planning, pervious pavements, shade trees, and the existence of water bodies in city areas are potential urban heat island mitigation strategies [25,26,27,28,29].

In addition to the absorption of solar energy, the accumulation of urban heat comes from the conversion of other energy. Human beings are the subjects of cities and other human activities, including natural respiration and industrial production, both of which play important roles in the formation of the thermal environment. Therefore, the urban thermal environment is related to population density, urban transportation, and industrial structure [15,19,30]. The air pollutants, products, and results of human activities also impact the urban thermal environment [31].

The scattered distribution of buildings with high rises can facilitate LST mitigation. Densely distributed buildings with low rise can easily become the largest heat source in a city [32,33]; we later explore possible limitations. In the current literature, scarce research has investigated influential factors of urban thermal environment on a microscale, especially regarding the shape, materials, and distributions of buildings. The influence of landscape configuration and diversity factors on LST is relatively weak and can be easily concealed by the influence of landscape components [32,34,35]. Nevertheless, the characteristics and distributions of buildings within a city are important factors that determine heat transfer. Unraveling how urban morphology influences urban thermal environment intensity is paramount given the human health consequences associated with the continued growth of urban populations in the future [36]. The local climate and landscape should be considered to eliminate urban thermal environment hazards in future urbanization processes [35].

One vital contribution of urbanization and industrialization is to circle a given number of buildings and structures and divide them as “closed” plots. Consequently, the effects of buildings and structures—within or between plots—force meteorological elements to vary at the community scale [37] and then form a microclimate. To learn the driving principle of formation of the internal thermal environment in “closed” plots, effectively improving the urban thermal environment and mitigating the harm to human health, taking commonly used vegetation and buildings as research objects, some studies are done in community-based microclimate: (1) the cooling effect of vegetation; (2) the impact of built-up features on the cooling effect; and (3) the reason and mechanism of the impact.

## 2. Materials and Methods

### 2.1. Study Area

Beijing—the center of national politics, culture, international exchanges, and technological innovation with high levels of urbanization—is the capital of the People’s Republic of China. Beijing’s climate is typically northern temperate semi-humid continental monsoon climate, i.e., hot and rainy in summer, cold and dry in winter, and short in spring and autumn. The frost-free period throughout the year is 180 to 200 days, and the western mountainous region is short. The seasonal distribution of precipitation is very uneven.

**Figure 1:**
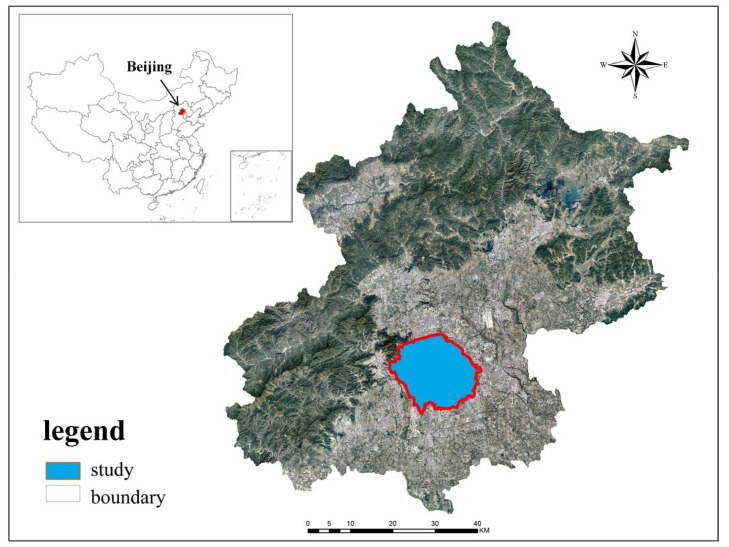
Location of built-up area in Beijing, China.

Because the distribution of buildings is compact and the phenomenon of urban heat islands is obvious, making it suitable for the research of the urban thermal environment, the area within the 5th ring road of Beijing was selected as the study area (Figure 1).

### 2.2. Data

Two main types of data—vector data and raster data—were used in this paper:

Vector data: To extract the building density and building height in the study area, the community outline data and building outline data of Beijing’s central urban area in 2017 were selected.

Raster data: To extract the NDVI and LST in the study area, we selected the Landsat 8 operational land imager and thermal infrared sensor (TIRS) images data (Table 1). The remote sensing image of the summer with a small amount of cloud was selected, which was downloaded from the USGS. To avoid the interference of rainfall, there was no rainfall on the day of the selected image and the days before and after. In order to make the research results more scientific, this article selected remote sensing images on 10 July 2017 for verification.

### 2.3. Research Methods

#### 2.3.1. Research Framework

**Figure 2:**
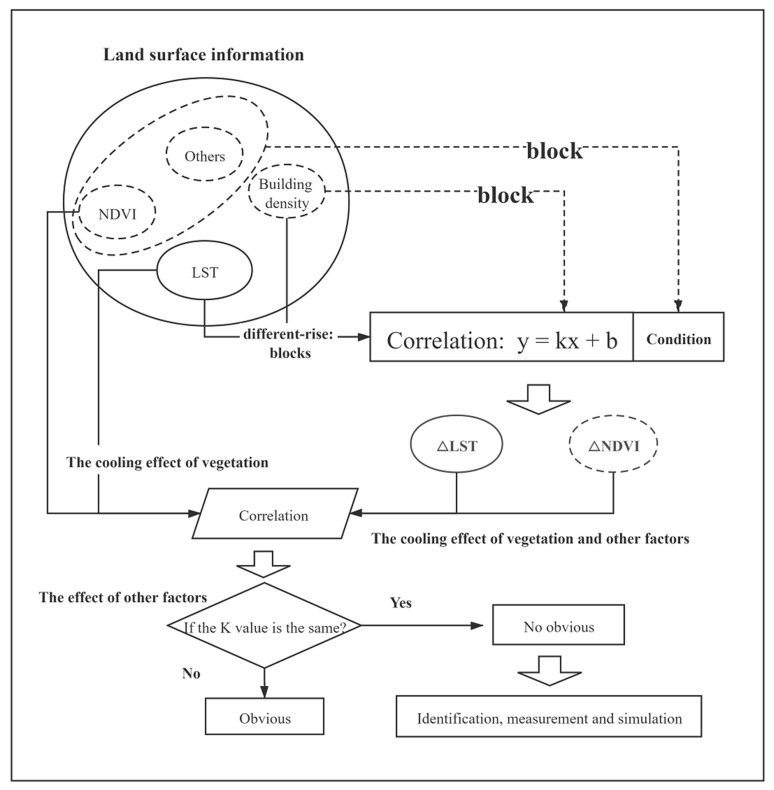
The research framework of impacts of built-up characteristics on the effect of the vegetation.

As one of the main elements of the urban environment, both buildings and vegetation had a great impact on land surface temperatures. Affected by urban planning, the distribution of buildings and vegetation within the city was mutually restricted; therefore, the research content of this article is divided into the following four parts (Figure 2): (1) matching the spatial distribution of vegetation and building features; (2) analyzing the cooling effect of vegetation; (3) examining the influence of buildings on the cooling effect of vegetation; and (4) analyzing, measuring, and simulating the impact mechanism of buildings.

#### 2.3.2. Surface Information

##### Calculation of Building Density

**Figure 3:**
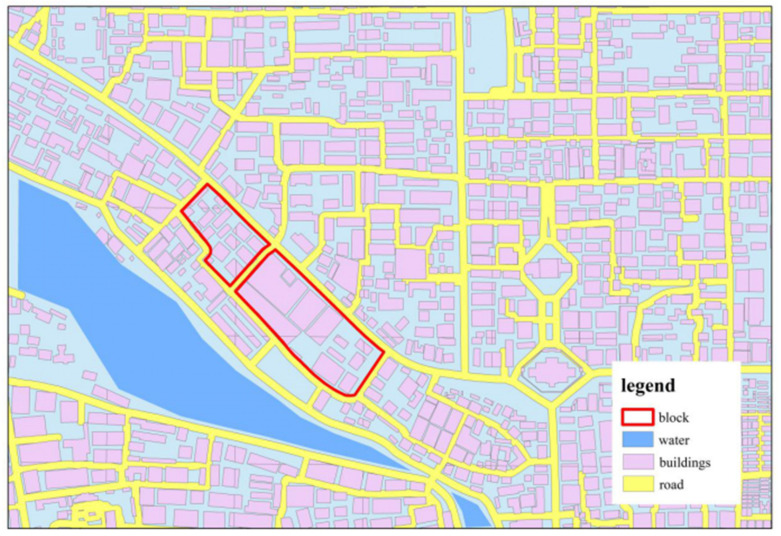
Part of the study area, the purple area, is the buildings; the study unit is a block composed of buildings, as shown in the red border area in the figure. The blue area is the water, and the yellow area is the road, which will be removed during the study.

Statistical units are the blocks formed by the community outline where the buildings are located, rather than traditionally fishing grids, for the reason that many complete buildings are divided into two or more statistical units by traditional fishing grids. Compared with the fishing grids, the blocks not only preserved the integrity of the building but also avoided the effects of roads. In addition, to avoid the impacts of water, all samples containing water were eliminated in the blocks (Figure 3).

The building density is the proportion of the area of the buildings within the block. The formula for calculating building density is as follows (1):

(1) $$d=\frac{\sum_{i=1}^{n}S_{i}}{S}$$

where d is the building density, $S_{i}$ is the area of the buildings within a block, and S is the total area of a block.

##### LST Retrieval

Estimation of land surface temperature (LST) from Landsat data requires a systematic methodology. The single-window method was used to retrieve LST in this study. The improved mono-window (IMW) algorithm was used as an efficient method for LST retrieval from the Landsat 8 TIRS Band 10 data. Validation of the IMW algorithm using the simulated datasets for various situations indicated that the LST difference between the retrieved and simulated ones was 0.67 K, on average [38].

##### Calculation of NDVI

The normalized difference vegetation index (NDVI) is a linear combination between the near-infrared band and red band, which is regarded as the basic index for measuring the “greenness” of the earth’s surface [7]. The use of optical remote sensing data in the computation of environmental indices of this region was hampered by the presence of clouds. There were two effective methods: the first algorithm was the automatic cloud removal method (ACRM), which employed a linear regression between the different spectral band sand the cirrus band; the second algorithm was an independent component analysis (ICA), which considered the noise (clouds) as part of independent components applied over the study area. After improving ACRM, the NDVI was computed using ACRM, which showed a better correlation than ICA with the MODIS NDVI product [39].

#### 2.3.3. The Cooling Effect of Vegetation

##### Matching of Vegetation and Building Characteristics

The area of green space is important for urban construction during urban planning, providing a comfortable environment for human beings and regulating the urban climate [40]. However, the distribution of buildings impacts the distribution of urban green space, where the building density is higher, and the vegetation is generally less distributed. Therefore, the building distribution could not be ignored when discussing the cooling effect of vegetation. Because of the height and density characteristics of the buildings, the NDVI in the sample points was classified and counted (Table A1 and Table A2 in Appendix A). We counted the NDVI value of the sample points in each group and selected the area within the quartile as the vegetation distribution of the group through box-plot analysis.

Therefore, we classified all samples according to different building heights and different building densities. The samples were divided into three major building height zones: low, medium and high-rise, and each zone was divided into 10 small zones according to different building density. Sample points in each area are used for the next analysis.

##### Correlation Analysis between NDVI and LST

Vegetation cover—an important factor for regulating the urban thermal environment—has a significant negative correlation with LST, and its cooling effects are closely related to its distribution [7,41]. We analyzed the correlation between NDVI and LST of sample points in different groups and then judged the cooling effect of vegetation under different building characteristics.

#### 2.3.4. The Impacts of Buildings on the Cooling Effect

With the development of urbanization, the phenomenon of the urban heat island has become more obvious, and the change of LST has a great relationship with a city’s environment. In order to analyze the impact of building characteristics on the cooling effect of vegetation, it is necessary to eliminate other factors in the study area.

##### The Relationship between Building Density and LST

Both building density and building height certainly affect LST. All blocks in the study areas were divided into different groups according to different building heights, including high-rise of 9 or more floors, middle-rise of 4–9 floors, and low-rise of 1–3 floors. Then, the building density and LST of all blocks were counted and input in SPSS software for correlation analysis.

##### Correlation between ΔNDVI and ΔLST

There was a strong linear correlation between building density and LST; the scatter plot of which showed a straight band distribution. The functional relationship was summarized as a straight line, y = kx + b. Therefore, the LST of blocks was estimated by bringing the building density value into y = kx + b. However, the environment in each block was different, and where the buildings, vegetation and other factors impacted LST. Therefore, the functional relationship between building density and LST was only a comprehensive relationship of all blocks. If the building density of a block was brought into the functional relationship, the result was likely to be different from the LST obtained by retrieving. We defined the difference between the retrieved LST and the calculated LST as ΔLST, where the inverted LST is obtained by inversion of remote sensing images, and the calculated LST is calculated by the functional relationship.

**Figure 4:**
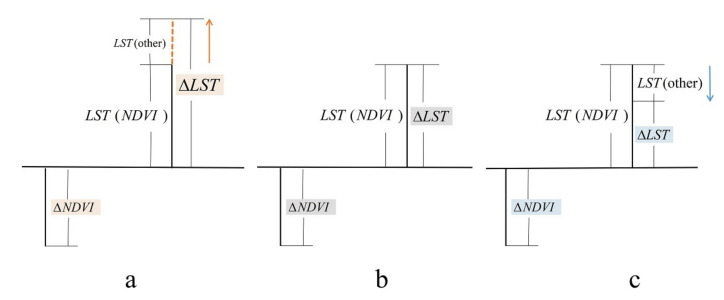
Analysis of the influence of other factors on the cooling of vegetation. (**a**) Other factors positively impact the cooling effect; (**b**) other factors negatively impact the cooling effect; (**c**) other factors have no impact on the cooling effect. Land surface temperature (LST) (normalized difference vegetation index (NDVI)) represents the LST impacted by NDVI. LST (other) represents the LST impacted by other factors.

In order to analyze the factors causing ΔLST, this paper used the average value of NDVI in the height groups as the vegetation environment of this group. For each individual block, the difference between the NDVI value and the NDVI average value indicates the difference in vegetation distribution. This paper assumed that the change of ΔLST was entirely caused by ΔNDVI and that the correlation between the two was the cooling effect of vegetation. Comparing this relationship with the cooling effect of vegetation, if the difference between the results was obvious, it meant that other factors had a greater impact on LST (Figure 4a,c). However, if the difference was relatively small, it meant that other factors could be ignored (Figure 4b).

(2) $$\Delta \mathrm{NDVI}={\mathrm{NDVI}}_{t}-{\mathrm{NDVI}}_{0}$$

(3) $$\Delta \mathrm{LST}={\mathrm{LST}}_{t}-{\mathrm{LST}}_{0}$$

where ${\mathrm{NDVI}}_{t}$ indicates the NDVI value of blocks, ${\mathrm{NDVI}}_{0}$ indicates the average NDVI value in the different building height zones, ΔNDVI indicates the difference between NDVI and NDVI0, ${\mathrm{LST}}_{t}$ indicates the true LST of blocks. ${\mathrm{LST}}_{0}$ represents the LST value calculated from the functional relationship, and ΔLST indicates the difference between ${\mathrm{LST}}_{t}$ and ${\mathrm{LST}}_{0}$.

In this article, if the hypothesis is valid, it means that other factors have little impact on the cooling effect of vegetation except for building density in the case of calculating the cooling effect of vegetation grouped by building density.

## 3. Results and Discuss

### 3.1. Surface Information

**Figure 5:**
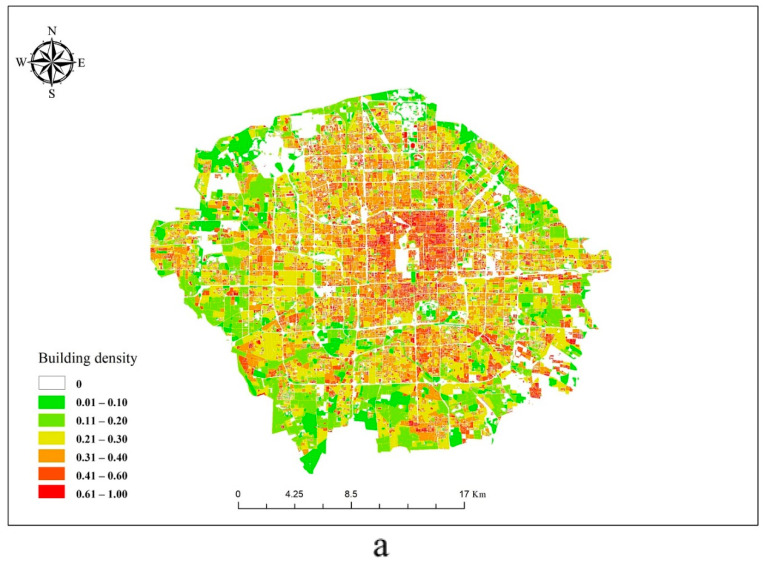

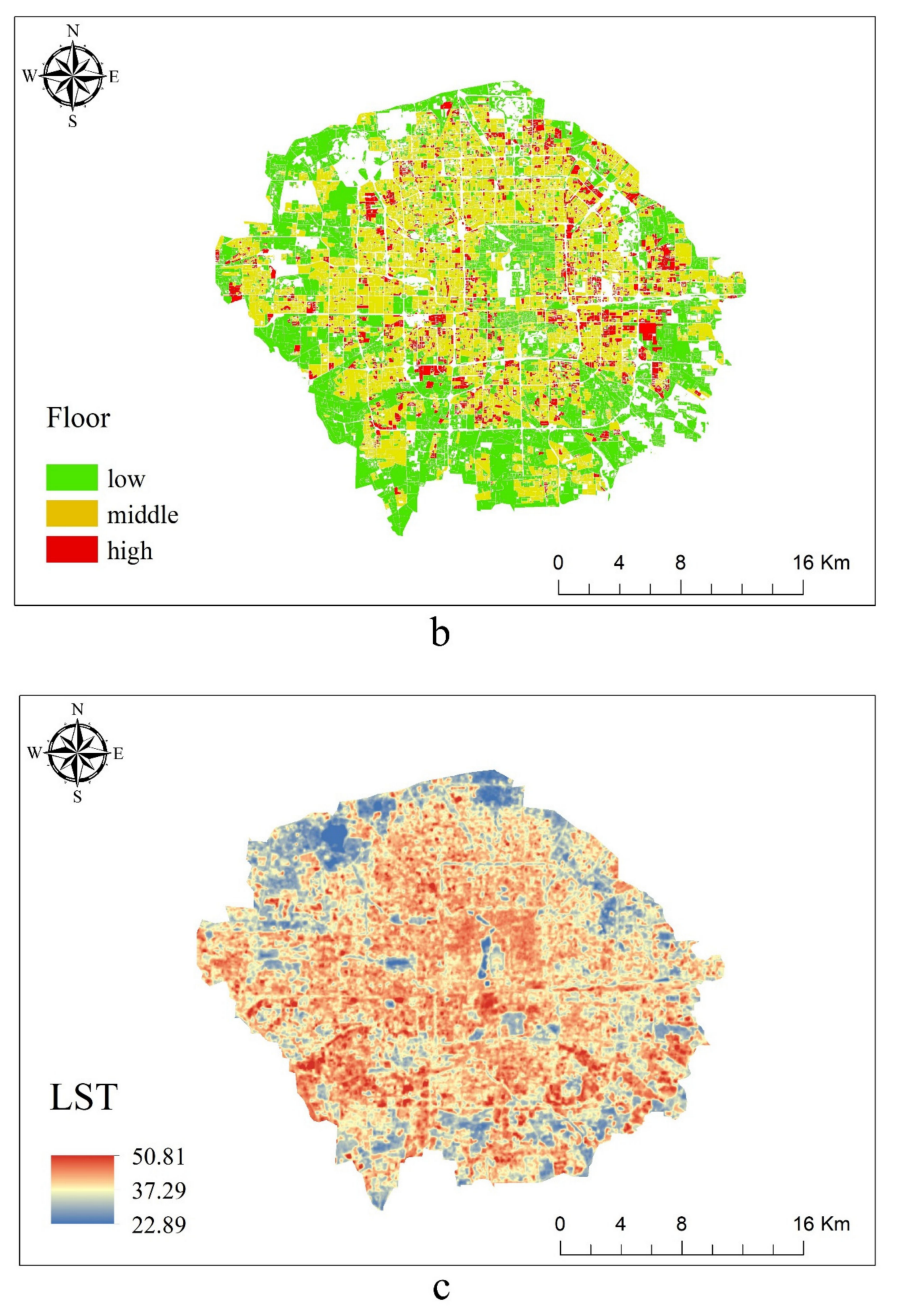

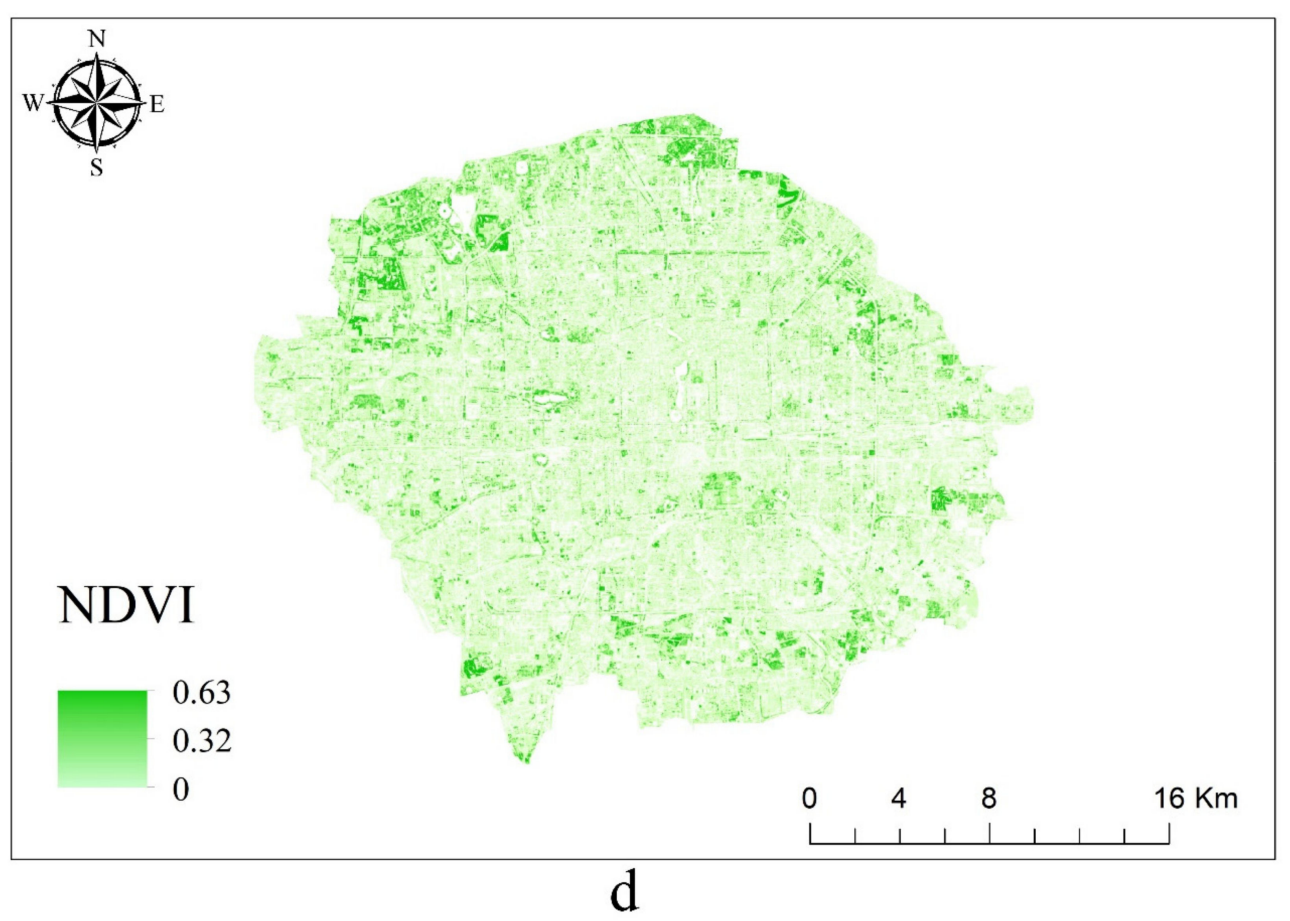
Surface information in the study area. (**a**) Building density, (**b**) building height, (**c**) LST and (**d**) NDVI.

The land surface information, including the spatial distribution of LST, building density, building height, and the NDVI index, were reported in the figure by ArcGIS 10.2 (Figure 5). Comparing the building density with the LST, it was found that the building density gradually decreased from the center to the periphery of the study area, and the LST maintained a synchronous change. The LST in the south was higher than the LST in the north, which may be related to the distribution of more water in the north (white area in Figure 5). Further, there were more train stations and airports in the south, which had a positive effect on LST [21]. Moreover, there were more low-floor buildings in the south of the study area and more middle- and high-floor buildings in the north. Building height had a certain negative correlation with the land surface temperature [42,43]. Comparing LST and NDVI, the LST of the region with high NDVI value was smaller, as shown in the blue region.

### 3.2. Distribution of Vegetation and Buildings

**Figure 6:**
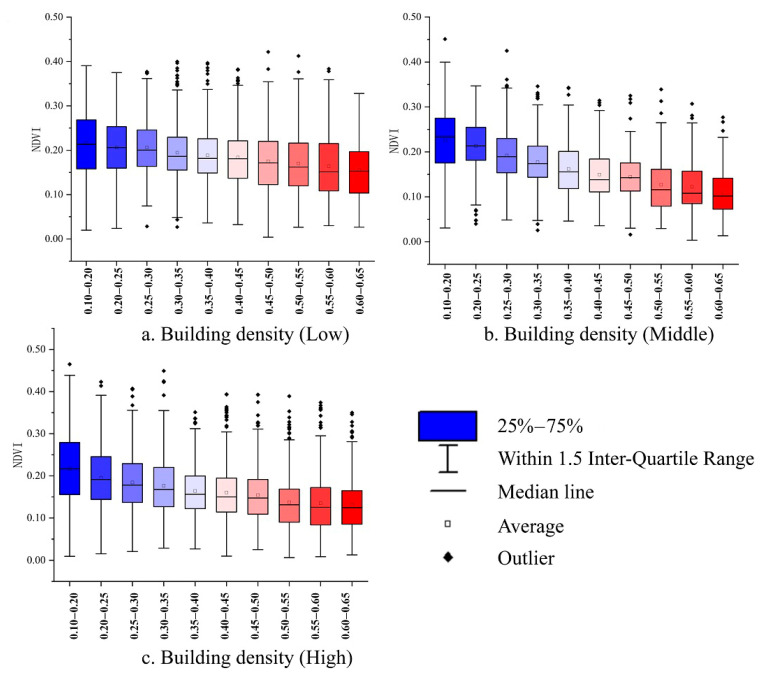
The matching of vegetation and building density under different building height groups. (**a**) Low-rise, (**b**) middle-rise and (**c**) high-rise.

The average value of NDVI for the low-rise building group in the study area was 0.166, the middle-rise building group was 0.186, and the high-rise building group was 0.163. In different building height groups, the distribution of vegetation decreased with the increase of building density. Among them, for middle-rise buildings, vegetation decreased the fastest, and the low-rise was the slowest (Figure 6).

### 3.3. The Cooling Effect of Vegetation and the Interference of Other Factors

#### 3.3.1. The Relationship between Building Density and LST

**Figure 7:**
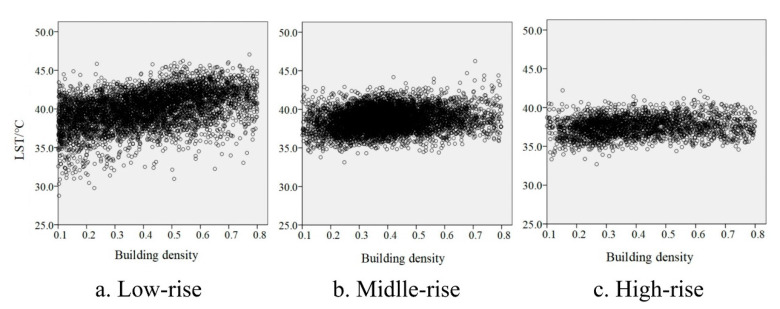
Scatter plot of correlation between building density and land surface temperature. (**a**) Low-rise: y = 5.608x + 37.683 (R² = 0.186, sig = 0.00); (**b**) middle-rise: y = 1.831x + 37.967 (R² = 0.030, sig = 0.00) and (**c**) high-rise: y = 1.207x + 37.068 (R² = 0.026, sig = 0.00), where x is the building density and y is LST.

The significant positive correlations between building density and LST appeared on different building height zones, and the slope of linear relationships gradually reduced from low-rise to high-rise (Figure 7).

#### 3.3.2. Comparison of Cooling Effect

Due to the significant negative linear correlation between NDVI and LST, the k and slope of the linear correlation were considered to be the cooling effect of vegetation on LST (Table A1 in Appendix A). K (the slope of the correlation between NDVI and LST) was approximately equal to Δk (the slope of the correlation between NDVI and LST), indicating that when grouped by building density, only building features and vegetation had a significant impact on surface temperature, and other factors could be ignored. Therefore, the cooling effect of the vegetation in the study area on the surface temperature was mainly affected by two factors: its own distribution characteristics and architectural characteristics.

### 3.4. Impact of Building Characteristics on Vegetation Cooling Effects

**Figure 8:**
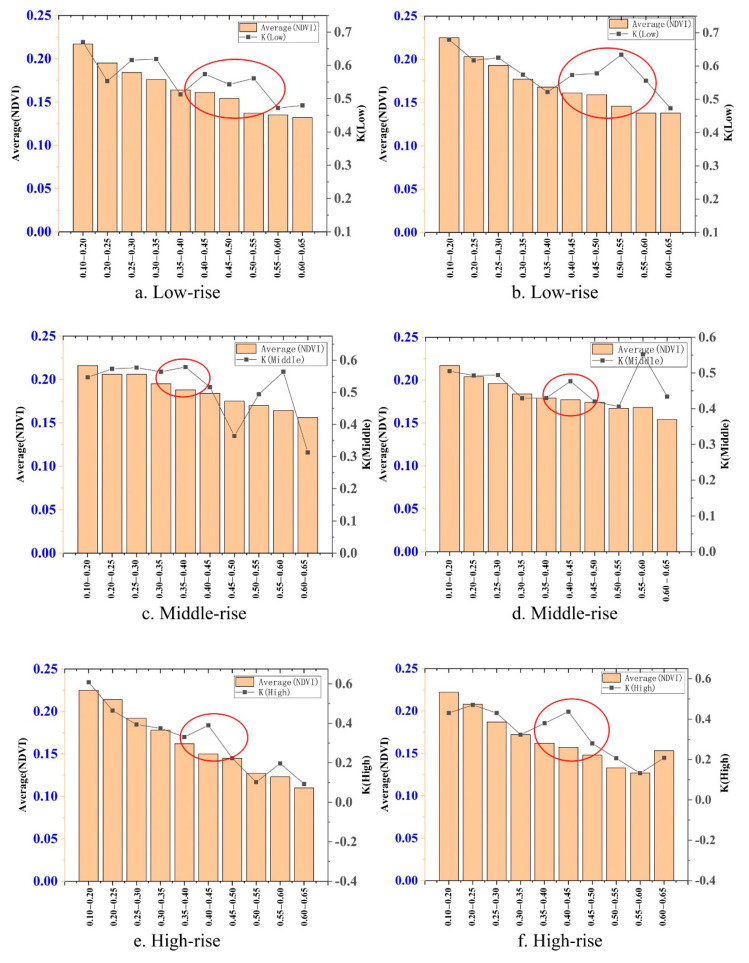
Comparison of the cooling effect and distribution of vegetation under different building densities in different building heights. (**a**) Low-rise, (**c**) middle-rise and (**e**) high-rise on 7 May 2017; (**b**) low-rise, (**d**) middle-rise and (**f**) high-rise on 10 July 2017.

In order to make the research results of the main image more credible. We chose another verification image to analyzed using exactly the same processing method as the main image and got the research results shown in Figure 8. Analyzed in the order of building density from low to high, the change trend of the cooling effect of vegetation (k) was mostly consistent with the change trend of distribution of vegetation (NDVI); however, there were also some differences, and the differences had a certain regular compared with the value of building density. It should be noted that the comparison of k and NDVI in this article was not for numerical values but for change trends.

#### Low-Rise Building Height Zone

When the building density was 0.4–0.5, Figure 8a,b both showed that the k(low) was higher than NDVI. In the range of 0.25–0.35, Figure 8a also showed that k(low) was higher than NDVI, and Figure 8b showed the same change trend between the two. Moreover, it was opposite to Figure 8a,b in the range of 0.55–0.6. There were few buildings (building density <0.35) in the low-rise building height zone of the study area; therefore, the difference in Figure 8a may be caused by fewer sampling points.

#### Middle-Rise Building Height Zone

Within the building density of 0.35–0.45, both Figure 8c,d showed that k(middle) was higher than the NDVI. When the building density ≥0.5, the k(middle) was occasionally higher than or lower than the NDVI in Figure 8c, while the k(middle) always was higher than NDVI in Figure 8d.

#### High-Rise Building Height Zone

The k(high) and NDVI in Figure 8e,f had the same changing trend. Only when the building density was 0.35–0.5, the k(high) was higher than NDVI.

Due to the limitation of the shooting period and cloud cover, the time of the two remote sensing images selected in this paper were 7 May 2017, and 10 July 2017. Moreover, the weather conditions should be kept as consistent as possible (Table 1). However, there were still two different conditions the one was the wind direction (the former was northwest wind, the latter was southwest wind), and the other was the temperature difference between the two. In this case, the same part between the two image results could be considered a reliable research result, and the different parts may be caused by different environmental conditions.

Therefore, when the building density <0.35, the change trend was the same for the cooling effect of vegetation and distribution of vegetation, indicating that building density had no effect on the cooling effect of vegetation. When the building density was 0.35–0.5, the cooling effect of vegetation was higher than the distribution of vegetation, indicating that building density enhanced the cooling effect of vegetation. However, when the building density >0.5 in the high-rise building zone, the change trend was the same for the cooling effect of vegetation and distribution of vegetation. In the middle- and low-rise building zone, the cooling effect of the vegetation may be higher or lower. This showed that when the building density >0.5, the cooling effect of vegetation was also impacted, but the impact did not have an obvious regular with building density.

According to the research in Section 3.5.2, when the building density ≥0.5, the cooling effect of vegetation was more affected by the spatial distribution of buildings. Moreover, the results of the two images were also different, which may be caused by different wind directions. For the same image, the same wind passed through blocks with different spatial distribution, the wind speed inside the buildings was different, while for the different images, wind with different wind directions passed through the same block. The wind speed inside the buildings was also different.

### 3.5. Measuring and Simulation

#### 3.5.1. Analysis of Possible Factor

The effect factors mainly include surface information, aboveground information, and environmental information in the city. Surface information includes vegetation, buildings, water bodies, and roads [1,2,44]. Aboveground information includes wind speed, population density, and human activity [31,42]. Environmental information includes season, elevation, slope, soil texture, and humidity [45].

Because the factor was related to the building characteristics, looking for the factor around the building density was effective. The urban expansion of a high-density downtown morphological pattern causes wind speed per year to trend downward [46]. The average wind speed was significantly negatively correlated with urban heat island intensity and was part of planning the distribution of buildings to ensure adequate ventilation indoors and outdoors [30,47]. In summary, wind speed can change the land surface temperature and is related to building density, which may be a factor.

#### 3.5.2. Simulation of the Factor

**Figure 9:**
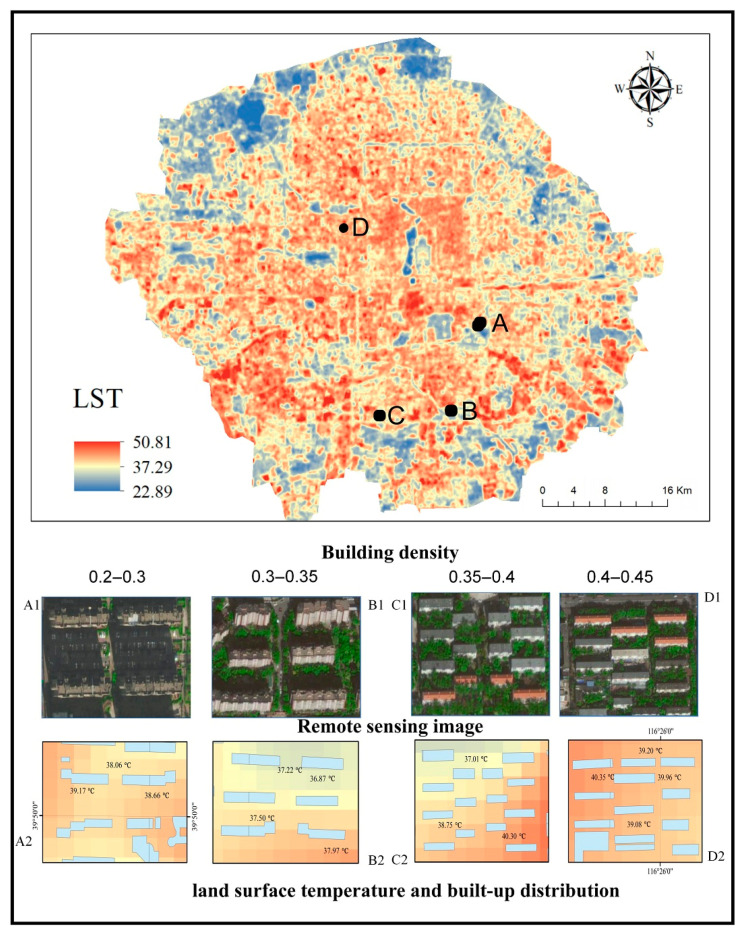
Four selected sample points in different building density: (A) 0.2–0.3, (B) 0.3–0.35, (C) 0.35–0.4 and (D) 0.4–0.45. (A1–D1) high-resolution imagery; (A2–D2) LST.

To verify the influence of wind speed on the cooling effect of vegetation and to explore how wind speed impacts cooling effects, this paper simulated the wind speed distribution characteristics of four various sample areas with land surface temperature. This paper selected four regions in the study area with the same vegetation distribution and different building densities, and their building heights belonged to the middle-rise (Figure 9). There was a positive correlation between building density and LST (Figure 7); however, the order of building density in Figure 9 was D > C > B > A, and the order of LST was D > A > C > B. Therefore, the reason for this result may be that the vegetation had a stronger cooling effect in the B and C compared to the A and D.

**Figure 10:**
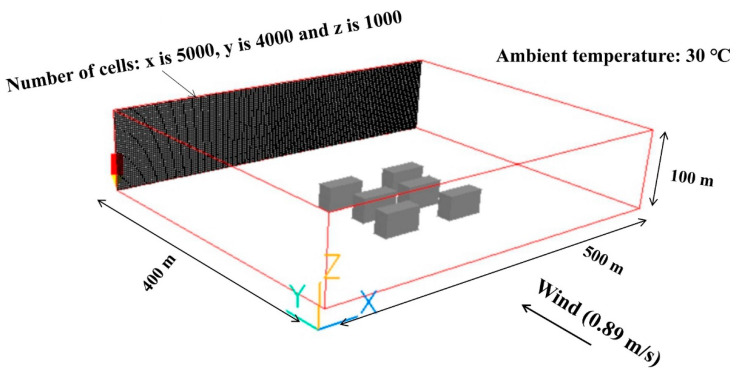
Computational domain, grid and boundary conditions of computational fluid dynamics (CFD) simulation in urban buildings.

**Figure 11:**
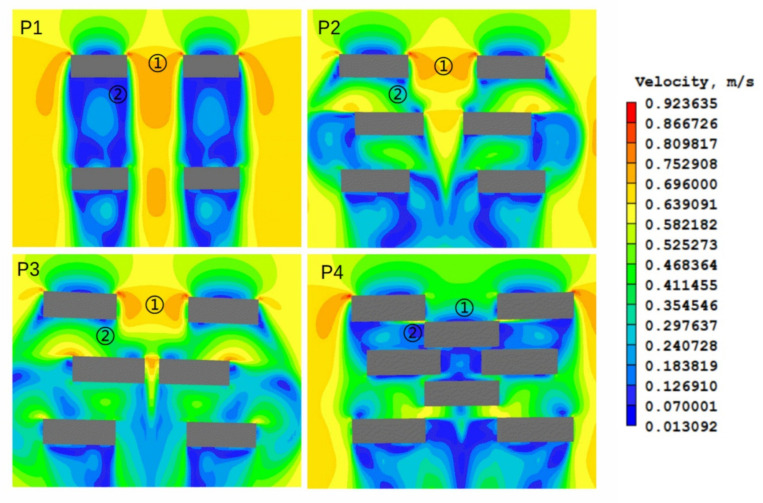
PHOENICS simulates the wind speed between buildings. (P1) 0.2–0.3, (P2) 0.3–0.35, (P3) 0.35–0.4 and (P4) 0.4–0.45.

This paper uses the computational fluid dynamics (CFD) method to simulate; the software used is PHOENICS. PHOENICS can accurately simulate the airflow phenomenon of the research object and accurately simulate the airflow, air quality, heat transfer, pollution and comfort of the ventilation system [48]. Since the complex urban environment existed around the simulation domain, a five-day continuous site observation was carried out on the thermal environment in the study area before the simulation. Measurement results for environmental conditions at the time of remote sensing image acquisition were used as the reference for setting boundary conditions. This paper cheese x, y and z of 500 m, 400 m and 100 m as the calculation geometry, and the number of cells were 5000, 4000, 1000, respectively. The calculation settings and boundary conditions are shown in Figure 10 [49,50]. The cross-sectional wind speed distribution at 1.5 m from the ground was extracted because the 1.5 m was the optimum height for the comfort of the human body due to the wind speed [51]. To compare and analyze the effects of the distance between buildings on the wind speed, points 1 and 2 were selected for analysis in the simulation diagram (Figure 11).

Point 1: The comparison of P1 and P2 showed that the smaller the building distance, the larger the wind speed. The comparison of P2, P3, and P4 showed that when the building spacing was the same, the distribution of buildings behind could impact the wind speed at point 1. However, the more crowded the building, the lower the wind speed. Moreover, the wind speeds of P2, P3, and P1 were not much different, showing that wind speed could be minimized only when there were a large number of buildings (Table 2).

Point 2: The comparison of P2, P3, and P1 showed that the uneven distribution of the building was employed to increase the wind speed inside the buildings. The comparison of P2, P3, and P4 showed that appropriate distribution of buildings was required; otherwise, too many buildings could cause wind speed to decrease or even stop (Table 2).

Therefore, the effects of buildings on wind speed mainly have two points: (1) the building distance impacted the wind speed, i.e., the smaller the distance, the greater the wind speed; (2) the uneven distribution of buildings impacted the wind speed, i.e., proper building distribution helped improve the ventilation inside the block. In the end, whether it is the building spacing or the uneven distribution of the building when the building density is large enough, the air circulation inside a building will be severely hindered, and wind speed will become smaller. When the building density is small, the wind speed changes synchronously; however, when the building density is large enough, the wind speed decreases. This feature is the same as that of a covered-up factor.

This analysis explains why the cooling effect of vegetation is stronger when the building density is 0.35–0.50. However, in the middle- and high-rise areas, when the building density reaches 0.5–0.6, the cooling effect of vegetation is very strong (Figure 8b,c,e). This article speculated that this might also be related to wind speed because a large number of examples have proven that it is easy to form a greater inter-building wind between closely adjacent tall buildings, which can also be called the street canyon effect. Of course, to prove this requires more scholars to conduct further research.

## 4. Conclusions

### 4.1. Main Achievements

Many studies have shown that LST was impacted by building characteristics and vegetation distribution, but the interaction between the factors has rarely been paid attention to. This paper analyzed the cooling effect of vegetation and studied the influence of building characteristics on the cooling effect. Through comparative research, this paper found that the cooling effect of vegetation was analyzed according to different building densities, and the interference of other influencing factors was small. The main influence on the cooling effect of vegetation was building characteristics.

The urban thermal environment was impacted by vegetation distribution and architectural characteristics. At the same time, there were also some factors that have not been discovered or proven in the city, which were easily covered up. This paper discovered the characteristics of wind speed changes hidden under the building features, as well as its impact on LST. The wind speed between buildings change with the increase of building density, and the changes in wind speed do impact the LST. A reasonable building density value can fully reflect the characteristics of urban ventilation and achieve the effects of alleviating urban heat islands. In addition, wind speed was not only impacted by the building density but also related to the spatial distribution of the building. A reasonable building structure will provide a powerful aid to alleviate the urban heat island. Through comparative analysis, this paper found that when the building density was 0.35–0.50, the cooling effect of vegetation was better. This conclusion provides powerful help for urban planning and mitigating the impact of the thermal environment on human health.

### 4.2. Limitations and Uncertainties

Due to the effects of the water body on the surrounding land surface temperature, the analysis of correlations between vegetation, building density, and land surface temperature were disturbed [19,34]. However, there were very few water bodies in the study area, and outliers were removed during correlation analysis. Thus, this paper minimized the interference effects of the above factors.

The wind speed between buildings was a highly susceptible factor and so real-time measurement was difficult. Therefore, the simulation was used to prove the change characteristics of wind speed between buildings. In this paper, only representative sample areas were selected for wind speed simulation, which could not fully express the relationship between wind speed and building features. We concluded that the wind speed was impacted by different building densities and building distributions through simulation experiments, yet uncertainty was still present. Therefore, we were only trying to reveal this problem, because there was no more support, we could not be sure that this complex urban issue was fully explained, but at least for our graphs and conclusions, we could confirm that this potential factor exists, and we also hope more scholars come to support our view.

### 4.3. Implications for Environmental Management

Urbanization is a global trend. Until now, more than 50% of the human population are situated in cities [23]. Thus, cities have become the main living environment for people, and, as such, it is significant to study the mechanism of the urban heat island. This research showed that the characteristics of buildings have an impact on the cooling effect of vegetation, and the specific performance was the cooling effect of wind speed. We found that good ventilation not only effectively avoided the accumulation of air pollutants in the city, but it improved the land surface temperature and brought residents a cooling temperature in the hot summer [8,9,10,11,12]. Whether focusing on sustainable development of the environment or human’s physical and mental health, urban ventilation is a necessary element to add to cities. This article determined the range of building density for optimal ventilation (Figure 11), which had a great reference value for urban planning.

In recent years, increasing ventilation corridors have been constructed. Ventilation is one of the important measures to alleviate urban heat islands [26]. Studying the effects of urban forms on ventilation was herein employed to somewhat avoid urban heat accumulation in urban planning. Nevertheless, it is difficult to directly associate architectural features with wind speed [47]. This article reflected the effect of wind speed on the surface temperature through the cooling effect of vegetation, which will provide ideas for other researchers.

## Figures and Tables

**Table 1:** Information on remote sensing images.

Type	Sensor	Landsat 8
Main	Acquisition date and satellite overpass time (IST)	7 May 2017, 9:52 a.m.
	Maximum air temperature (°C)	30
	Minimum air temperature (°C)	15
	Relative humidity (%)	15
	Cloud cover	0.01
	Condition	Fair
	Wind speed (m/h)	0.89
	Wind direction	Northwest
Verification	Acquisition date and satellite overpass time (IST)	10 July 2017, 9:53 a.m.
	Maximum air temperature (°C)	36
	Minimum air temperature (°C)	19
	Relative humidity (%)	38
	Cloud cover	0.01
	Condition	Fair
	Wind speed (m/h)	0.89
	Wind direction	Southwest

**Table 2:** Wind speed simulation table between buildings.

Scene	Distance between Buildings in the First Row (m)	Sampling Point	Wind Speed (m/s)
P1	72	①	0.722
		②	0.083
P2	90	①	0.694
		②	0.361
P3	90	①	0.667
		②	0.389
P4	90	①	0.333
		②	0.166

## Appendix A

**Table A1:** The distribution and cooling effect of vegetation under different building densities in different building height groups on 7 May 2017. K represents the slope of the correlation between NDVI and LST; ΔK represents the slope of the correlation between ΔNDVI and ΔLST.

Building Floor	Building Density	0.1–0.2	0.2–0.25	0.25–0.3	0.3–0.35	0.35–0.4	0.4–0.45	0.45–0.5	0.5–0.55	0.55–0.6	0.6–0.65
Low	25%	0.155	0.144	0.137	0.127	0.122	0.114	0.109	0.090	0.083	0.082
	75%	0.279	0.245	0.229	0.221	0.200	0.195	0.192	0.169	0.172	0.165
	Average	0.217	0.195	0.184	0.176	0.164	0.161	0.154	0.137	0.135	0.132
	K	0.670	0.553	0.616	0.619	0.513	0.574	0.543	0.561	0.472	0.480
	R²	0.449	0.305	0.380	0.383	0.264	0.330	0.295	0.315	0.223	0.231
	ΔK	0.665	0.549	0.616	0.616	0.514	0.574	0.543	0.557	0.472	0.480
	R²	0.442	0.301	0.379	0.379	0.265	0.330	0.295	0.311	0.223	0.230
Middle	25%	0.158	0.160	0.164	0.155	0.149	0.137	0.123	0.120	0.108	0.103
	75%	0.270	0.253	0.246	0.229	0.226	0.221	0.220	0.216	0.215	0.198
	Average	0.216	0.206	0.206	0.195	0.188	0.184	0.175	0.170	0.164	0.156
	K	0.547	0.573	0.577	0.564	0.579	0.516	0.364	0.494	0.564	0.313
	R²	0.300	0.327	0.332	0.318	0.335	0.267	0.132	0.244	0.319	0.098
	ΔK	0.548	0.573	0.577	0.564	0.578	0.517	0.363	0.494	0.564	0.315
	R²	0.300	0.328	0.333	0.318	0.334	0.267	0.132	0.244	0.318	0.099
High	25%	0.175	0.181	0.154	0.143	0.118	0.111	0.113	0.079	0.085	0.073
	75%	0.275	0.255	0.230	0.213	0.201	0.184	0.175	0.162	0.157	0.142
	Average	0.225	0.214	0.192	0.178	0.162	0.150	0.145	0.127	0.123	0.110
	K	0.608	0.465	0.394	0.375	0.331	0.390	0.223	0.102	0.197	0.093
	R²	0.370	0.217	0.155	0.141	0.110	0.152	0.050	0.010	0.039	0.009
	ΔK	0.609	0.465	0.394	0.375	0.331	0.390	0.223	0.100	0.196	0.092
	R²	0.371	0.216	0.155	0.141	0.110	0.152	0.050	0.010	0.039	0.009

**Table A2:** The distribution and cooling effect of vegetation under different building densities in different building height groups on 10 July 2017. K represents the slope of the correlation between NDVI and LST.

Building Floor	Building Density	0.1–0.2	0.2–0.25	0.25–0.3	0.3–0.35	0.35–0.4	0.4–0.45	0.45–0.5	0.5–0.55	0.55–0.6	0.6–0.65
Low	25%	0.161	0.154	0.15	0.136	0.126	0.119	0.118	0.1	0.091	0.091
	75%	0.285	0.249	0.233	0.216	0.2	0.195	0.195	0.174	0.174	0.169
	Average	0.225	0.203	0.193	0.177	0.168	0.161	0.159	0.146	0.138	0.138
	K	0.679	0.617	0.625	0.574	0.522	0.573	0.578	0.634	0.556	0.473
	R²	0.461	0.381	0.391	0.329	0.273	0.328	0.334	0.402	0.310	0.224
Middle	25%	0.168	0.167	0.160	0.151	0.139	0.132	0.13	0.118	0.111	0.097
	75%	0.264	0.243	0.229	0.212	0.214	0.216	0.212	0.211	0.214	0.2
	Average	0.217	0.204	0.196	0.184	0.179	0.177	0.174	0.167	0.168	0.154
	K	0.505	0.493	0.494	0.429	0.430	0.477	0.42	0.406	0.552	0.434
	R²	0.255	0.179	0.244	0.184	0.185	0.228	0.177	0.164	0.304	0.189
High	25%	0.179	0.169	0.147	0.131	0.122	0.112	0.108	0.092	0.088	0.095
	75%	0.270	0.245	0.223	0.207	0.197	0.195	0.177	0.165	0.169	0.192
	Average	0.222	0.208	0.187	0.172	0.162	0.157	0.148	0.133	0.127	0.153
	K	0.43	0.470	0.430	0.323	0.379	0.437	0.279	0.206	0.132	0.208
	R²	0.160	0.221	0.185	0.104	0.144	0.191	0.078	0.042	0.017	0.043
